# Childbearing in women diagnosed with cancer during reproductive age

**DOI:** 10.1111/aogs.70071

**Published:** 2025-10-27

**Authors:** Giovanna Esposito, Anna Cantarutti, Matteo Franchi, Fedro Alessandro Peccatori, Giovanna Scarfone, Eva Negri, Giovanni Corrao, Fabio Parazzini, Carlo La Vecchia

**Affiliations:** ^1^ Department of Clinical Sciences and Community Health, Dipartimento di Eccellenza 2023–2027 University of Milan Milan Italy; ^2^ Fondazione IRCCS Ca' Granda Ospedale Maggiore Policlinico Milan Italy; ^3^ Unit of Biostatistics, Epidemiology and Public Health, Department of Statistics and Quantitative Methods University of Milano‐Bicocca Milan Italy; ^4^ National Centre for Healthcare Research and Pharmacoepidemiology Milan Italy; ^5^ Department of Gynecology European Institute of Oncology, IEO, IRCCS Milan Italy; ^6^ Obstetrics and Gynecology Unit Fondazione IRCCS Ca' Granda Ospedale Maggiore Policlinico Milan Italy; ^7^ Department of Medical and Surgical Sciences University of Bologna Bologna Italy; ^8^ Emeritus Professor of Medical Statistics University of Milan‐Bicocca Milan Italy

**Keywords:** cancer, childbearing, fertility preservation, oncofertility, reproductive age

## Abstract

**Introduction:**

Fertility after cancer represents a growing clinical concern. This study assessed childbearing outcomes among women diagnosed with cancer during reproductive age between 2012 and 2017 in Lombardy, the largest region in Italy.

**Material and Methods:**

Women aged 15–45 years with a primary diagnosis of cancer recorded in hospital discharge records from regional healthcare databases were selected. Each woman diagnosed with cancer was matched with up to five cancer‐free women of the same age at diagnosis. The cumulative probability of childbirth up to December 31, 2022 was estimated using the Kalbfleisch–Prentice cumulative incidence function estimator. Cox regression models were used to estimate the cause‐specific hazard ratios (HRs) and the 95% confidence intervals (CIs) of childbirth according to the cancer diagnosis. Furthermore, in the group of cancer survivors, exposure to antineoplastic treatment was considered and included in the model as a time‐dependent covariate. Finally, a log‐binomial regression model was used to assess the association between antineoplastic therapy and medically assisted reproduction.

**Results:**

A total of 13,877 women were diagnosed with cancer at reproductive age during the study period (1.16 per 1000 person‐years). The cumulative probability of childbirth was lower among women diagnosed with cancer compared to cancer‐free women across all age groups: 31.4% vs 32.2% (*p* = 0.02) among those diagnosed under 30, 13.3% vs 22.7% (*p* < 0.01) among those aged 30–39, and 0.8% vs 1.6% (*p* < 0.01) among those aged 40 and over. The corresponding HRs were 0.93 (95% CI: 0.83–1.05), 0.58 (95% CI: 0.53–0.64), and 0.52 (95% CI: 0.40–0.68). When analyses were stratified by time since diagnosis, the reduced probability among cancer survivors was confirmed to be significant only within the first 5 years after diagnosis, also for younger individuals. Antineoplastic treatment was associated with a reduced probability of subsequent birth (HR = 0.46, 95% CI: 0.39–0.52). Moreover, the therapy was positively associated with medically assisted reproduction (RR = 1.71, 95% CI: 1.14–2.56).

**Conclusions:**

The probability of childbearing was reduced within the first 5 years of diagnosis, regardless of the patient's age. A more pronounced reduction was observed in women diagnosed after the age of 30. Age and antineoplastic therapy were key factors in determining childbearing in women diagnosed with cancer.

## Graphical abstract

Compared to cancer‐free women, the probability of childbearing within 5 years of a cancer diagnosis was reduced, particularly among those diagnosed over the age of 30. Age at diagnosis and antineoplastic treatment were key factors, with treatment also increasing the use of assisted reproduction.

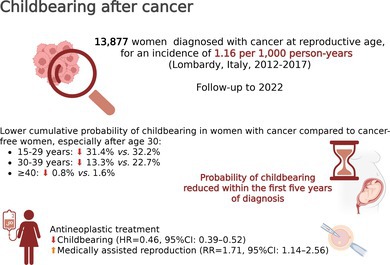

**Key message**

Among women diagnosed with cancer during their reproductive age, childbearing was reduced, especially in older patients and those receiving antineoplastic treatment.

## 1 INTRODUCTION

Approximately one million new cases of cancer are diagnosed in women of childbearing age (15–49 years) worldwide each year.^1^ Reproductive health is a major concern for young women diagnosed with cancer, as they may face many fertility‐related challenges. Biological, psychological, and social factors influence the likelihood of childbearing and the choices of women. The potential for infertility is a primary source of anxiety, since oncological treatments may reduce fertility.^2^, ^3^, ^4^ Chemotherapy and radiotherapy can damage the ovaries, leading to the loss of ovarian follicles or premature ovarian failure,^5^, ^6^ thus hampering conception. The fear of disease recurrence may influence the decision to seek pregnancy.^7^ Moreover, facing the possibility of infertility can lead to anxiety and depression. For women who have not yet started a family, the cancer diagnosis can influence and complicate decisions about pregnancy and contraception.^8^

Breast cancer is one of the most common neoplasms among women of reproductive age, accounting for approximately 10%–15% of all breast cancer cases.^8^ However, its incidence varies depending on the age group and population considered. Being diagnosed at a young age is an adverse prognostic factor,^9^ and as a result, the majority of young women are offered adjuvant chemotherapy and/or hormonal therapy. Tumors in this population are more likely to be aggressive, leading to the frequent prescription of intensive regimens such as multi‐agent chemotherapy. These treatments can adversely affect ovarian function and fertility, either through the direct gonadotoxic effects of chemotherapy or by requiring a delay in conception until the completion of adjuvant therapy. Other cancers, like cervical and thyroid cancer, and some types of lymphomas, are common in young women.

This study aimed to assess the probability of subsequent childbirth among women of reproductive age diagnosed with cancer in Lombardy, Italy, between 2012 and 2017, with a focus on the most prevalent cancer sites. Specifically, it investigated potential differences in reproductive chances between cancer survivors and a matched cancer‐free cohort, and evaluated the association between exposure to antineoplastic treatments and the probability of subsequent childbirth.

## 2 MATERIAL AND METHODS

### 2.1 Data sources and study population

We conducted a population‐based cohort study using administrative data from regional healthcare databases in Lombardy, Italy's largest region, with nearly 10 million inhabitants. The National Health Service (NHS) in Italy covers the entire population. In Lombardy, the NHS has been linked to an automated database system since 1997. For this study, the following databases were consulted: the NHS archive of beneficiaries, the hospital discharge form [scheda di dimissione ospedaliera (SDO)], the certificate of delivery assistance [certificate di assistenza al parto (CedAP)], the outpatient specialist and diagnostic services registry, and the registry of the prescriptions of innovative and expensive drugs administered in hospital on an outpatient basis or distributed for home therapies (File F).

We identified women aged 15–45 with at least one hospital discharge form reporting a diagnosis of cancer between January 1, 2012 and December 31, 2017, and resident in the Lombardy Region. To ensure the inclusion of only newly diagnosed cases, prevalent cases were excluded. Prevalent cases were defined as women with a history of cancer (with at least one cancer‐related hospital discharge) within the 5 years preceding their cancer diagnosis (between 2012 and 2017). Each woman diagnosed with cancer was matched with up to five cancer‐free women of the same age at diagnosis identified from the NHS archive of beneficiaries. The selection process was performed in November 2024.

### 2.2 Outcome of interest

To determine whether a birth occurred after the cancer diagnosis (or, for the control group, the date of diagnosis of the matched cancer), we considered all deliveries recorded in the CedAP database from January 1, 2012 to December 31, 2022. Specifically, we analyze only pregnancies that resulted in delivery at 22 weeks of gestation or later, as recorded in the CedAP database. Pregnancies ending before 22 weeks (i.e., miscarriages or induced abortions) are not captured in the CedAP and were therefore excluded from our analysis. Only births whose conception was after the date of cancer diagnosis were taken into account.

### 2.3 Antineoplastic treatment exposure

Antineoplastic treatment was defined by the presence of at least one of the following: a hospital discharge diagnosis or procedure related to antineoplastic therapy, an outpatient visit indicating antineoplastic treatment, or an inpatient drug prescription recorded for antineoplastic drug use (ATC code: L01, including alkylating agents, antimetabolites, plant alkaloids and other natural products, cytotoxic antibiotics and related substances, protein kinase inhibitors, monoclonal antibodies and antibody drug conjugates, other neoplastic agents). A woman was considered treated if antineoplastic therapy was administered in the period from 1 year before the cancer diagnosis to the end of follow‐up.

### 2.4 Statistical analysis

The incidence rate of cancer in the fertile age group, with the corresponding 95% confidence interval (CI), was calculated as the number of cancer cases per 1000 person‐years. Person‐years were calculated using the NHS registry of beneficiaries. For each calendar year between 2012 and 2017, the number of women aged 15–45 was determined based on their year of birth. Incidence rates were also computed separately for the three most common cancers (i.e., breast, thyroid, and lymphomas). Differences in the age distribution of the three most common cancers were tested using the chi‐squared test (for age categories, i.e., 15–29, 30–39, 40–45 years) and one‐way ANOVA (for average ages).

The cumulative probability of childbirth among women diagnosed with cancer during their reproductive years and the matched cancer‐free women, stratified by age at cancer diagnosis, was estimated using the cumulative incidence function to account for the competing risk of death according to the Kalbfleisch–Prentice method. This was repeated across the most common cancer sites. Each woman accumulated person‐years of follow‐up from cancer diagnosis until the earliest of the following: childbirth date, exit from regional healthcare coverage, death, or December 31, 2022. Women were censored if they moved out of the region or did not have a birth by the end of follow‐up (December 31, 2022). Gray's test was used to compare cumulative incidence curves between cancer survivors and cancer‐free women.

Cox regression models were fitted to estimate the cause‐specific hazard ratios (HRs) and the corresponding 95% CIs of giving birth according to the presence of a cancer diagnosis in strata of age. We used age as the time scale to account for the natural decline in fertility associated with aging, setting the starting age at the time of cancer diagnosis and the ending age at either the time of giving birth or censoring. To address potential time‐varying effects of cancer on childbearing, we additionally performed analyses stratified by time since diagnosis (i.e., ≤5 and >5 years).

Subsequently, Cox regression models were also fitted to estimate the HR and the corresponding 95% CI of giving birth in cancer survivors according to antineoplastic treatment (treated as a time‐dependent variable). The models were repeated in the subsets of the most common neoplasms.

Finally, in the subgroup of women who gave birth after a cancer diagnosis, we identified births occurring after the use of medically assisted reproduction. The association between receiving antineoplastic therapy and medically assisted reproduction was assessed using a log‐binomial regression model adjusted for age at delivery. The relative risk (RR) and corresponding 95% CI for medically assisted reproduction according to antineoplastic therapy were reported.

All these data were handled anonymously. Ethical approval is not required in Italy for this study. All analyses were performed using the Statistical Analysis System Software (version 9.4; SAS Institute, Cary, NC, USA).

## 3 RESULTS

**FIGURE 1:**
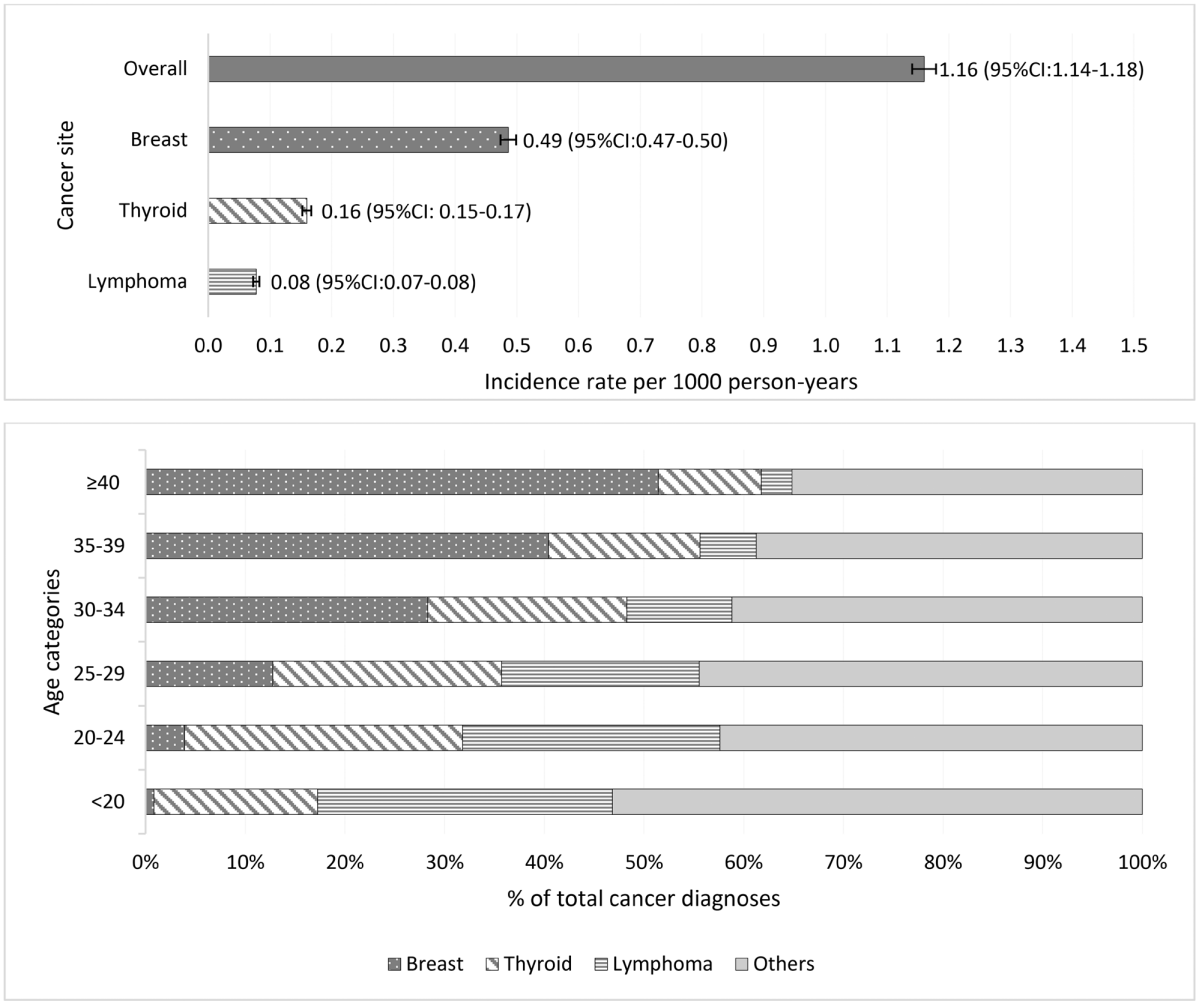
Incidence rate and 95% confidence intervals of cancer diagnosed in reproductive age, overall and according to cancer site (upper panel). Distribution of cancer sites according to age at diagnosis (lower panel). Lombardy (Italy), 2012–2017.

We identified 13 877 women diagnosed with cancer at reproductive age during the study period, corresponding to an incidence rate of 1.16 (95% CI: 1.14–1.18) per 1000 person‐years. The majority of the patients (56.9%) were aged 40 or over, and 6.5% had a pregnancy that resulted in a birth following a cancer diagnosis. The incidence rates of the most diagnosed cancers (i.e., breast and thyroid cancers, and lymphomas) are shown in the upper panel of Figure 1.

The distribution of cancer sites varied considerably by age (*p* < 0.01) (Figure 1, lower panel). Lymphomas were the most frequent (29.6% of all tumors) in very young women (aged 15–19 years) and less common at later ages (≥40 years) (3.1%). Other tumors diagnosed in very young women were those of the nervous system (11.8%), skeletal system (9.3%), and leukemia (8.8%). Breast cancer accounted for half of the diagnoses (51.5%) in older women (≥40 years), 40.4% in those aged 35–39 years, 28.3% in those aged 30–34 years, and less than 1.0% in the youngest ones. The average age (SD, standard deviation) at diagnosis for breast cancer, thyroid cancer, and lymphomas was 40.7 (4.1) years, 36.3 (7.2) years, and 31.9 (8.8) years (*p* < 0.01), respectively. The distribution of all cancer sites by age group is provided in Table 1.

**TABLE 1:** Distribution of sites by age at diagnosis in the 13 877 cancer cases in reproductive age. Lombardy (Italy), 2012–2017.

Site	Age at cancer diagnosis		
	15–29	30–39	≥40
	*n* (%)	*n* (%)	*n* (%)
Breast	109 (7.3)	1635 (36.5)	4063 (51.5)
Thyroid	341 (22.8)	751 (16.7)	814 (10.3)
Lymphoma	358 (23.9)	326 (7.3)	245 (3.1)
Metastases	65 (4.3)	242 (5.4)	387 (4.9)
Cervix	39 (2.6)	289 (6.4)	272 (3.4)
Ovary	65 (4.3)	147 (3.3)	265 (3.4)
Colon‐rectum	32 (2.1)	126 (2.8)	261 (3.3)
Skin	32 (2.1)	136 (3.0)	259 (3.3)
Endometrium	12 (0.8)	63 (1.4)	174 (2.2)
Nervous system	118 (7.9)	105 (2.3)	125 (1.6)
Leukemia	88 (5.9)	80 (1.8)	106 (1.3)
Melanoma	33 (2.2)	73 (1.6)	106 (1.3)
Lung	13 (0.9)	48 (1.1)	106 (1.3)
Kidney	9 (0.6)	52 (1.2)	103 (1.3)
Skeleton	83 (5.6)	70 (1.6)	102 (1.3)
Head and neck	26 (1.7)	80 (1.8)	91 (1.2)
Urinary tract	10 (0.7)	37 (0.8)	73 (0.9)
Stomach	2 (0.1)	41 (0.9)	70 (0.9)
Pancreas	6 (0.4)	29 (0.7)	61 (0.8)
Liver	8 (0.5)	16 (0.4)	19 (0.2)
Other sites	40 (2.7)	130 (2.9)	183 (2.3)
Not defined	7 (0.5)	9 (0.2)	11 (0.1)

*Note*: Percentages are calculated within columns.

**FIGURE 2:**
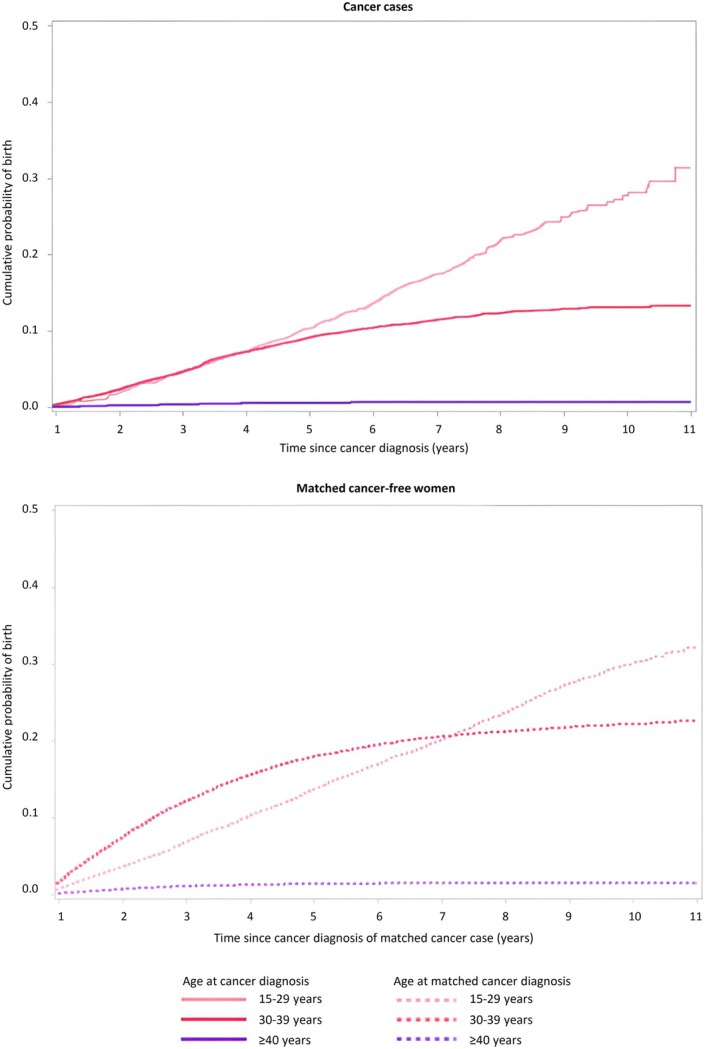
Cumulative birth probability in women diagnosed with cancer in reproductive age and matched cancer‐free women according to age at cancer diagnosis. Upper panel depicts results for cancer cases and is represented with a solid line, while the lower panel on the right shows results for matched cancer‐free women and is represented with a dashed line.

Figure 2 shows the cumulative probability of childbirth for women diagnosed with cancer during their reproductive years, compared to women without a cancer diagnosis, stratified by age at cancer diagnosis (i.e., 15–29, 30–39, ≥40 years). The median follow‐up period was 7.3 years (IQR: 5.5–9.1 years) among women with a cancer diagnosis and 7.7 years (IQR: 6.0–9.4) among matched cancer‐free women. The cumulative probability of birth was consistently lower among women diagnosed with cancer compared to cancer‐free women across all age groups: 31.4% vs 32.2% (*p* = 0.02) among those diagnosed before the age of 30, 13.3% vs 22.7% (*p* < 0.01) among those aged 30–39, and 0.8% vs 1.6% (*p* < 0.01) among those aged 40 and over.

**FIGURE 3:**
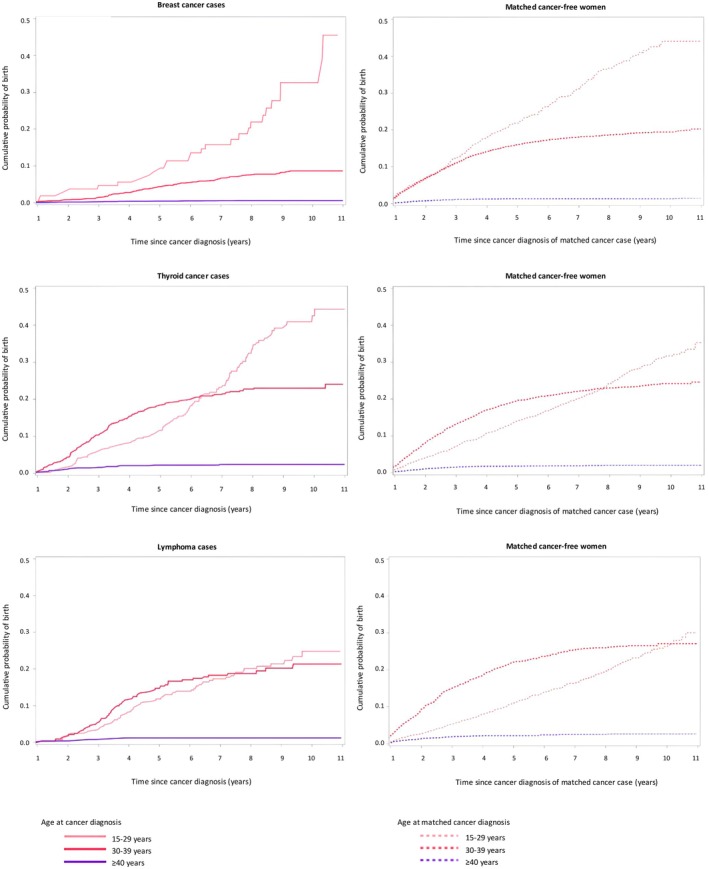
Cumulative birth probability in women diagnosed with cancer in reproductive age and matched cancer‐free women according to age at cancer diagnosis and cancer site. Panels on the left depict results for cancer cases and are represented with a solid line, while panels on the right show results for matched cancer‐free women and are represented with a dashed line.

Figure 3 provides the cumulative birth probabilities by cancer site. For women aged 15–29 years at diagnosis, the cumulative probability of childbirth was 45.5% for breast, 44.3% for thyroid cancers, and 24.8% for lymphomas, compared to 44.0% (*p* = 0.01), 35.3% (*p* = 0.02), and 30.0% (*p* = 0.83), respectively, in the matched cancer‐free controls. For those aged 30–39 years at diagnosis, the cumulative probability was 8.6% for breast, 24.1% for thyroid cancers, and 21.4% for lymphomas, with corresponding probabilities of 20.2% (*p* < 0.01), 24.6% (*p* = 0.54), and 27.1% (*p* = 0.01) in the matched cancer‐free controls. In the oldest group, aged 40 years or over at diagnosis, childbirth probabilities were markedly higher for breast cancer, with values of 0.6% vs 1.5% (*p* < 0.01) in matched cancer‐free counterparts. No significant differences were observed for thyroid cancer and lymphomas, with cumulative probabilities of 2.4% and 1.2%, respectively, compared to 2.0% (*p* = 0.50) and 2.7% (*p* = 0.21) in the cancer‐free group.

Table 2 provides the HRs for having a birth following a cancer diagnosis during reproductive age, both overall and stratified by major cancer types. No significant reduction in the probability of childbirth was observed among women diagnosed before the age of 30 years (HR = 0.93, 95% CI: 0.83–1.05). However, a significantly lower probability of childbirth was observed for those diagnosed between 30 and 39 years (HR = 0.58, 95% CI: 0.53–0.64), and for women diagnosed at age 40 or above (HR = 0.52, 95% CI: 0.40–0.68). When we stratified by cancer type, the greatest reduction in the probability of childbirth was observed among breast cancer survivors, particularly in the 30–39 age group (HR = 0.37, 95% CI: 0.31–0.45). In contrast, there was no overall reduction among thyroid cancer survivors, and an increased probability of childbirth was observed in women diagnosed before the age of 30 (HR = 1.28, 95% CI: 1.03–1.59). A moderate reduction in childbirth probability was observed among lymphoma survivors in the 30–39 age group (HR = 0.72, 95% CI: 0.55–0.94), whereas no significant differences were observed among those diagnosed at younger ages. When analyses were stratified by time since diagnosis, the reduced probability of childbirth among cancer survivors was confirmed to be significant within the first 5 years after diagnosis, also for younger, but not thereafter (Table S1). This pattern was consistent across all age groups and cancer types, indicating that the impact of cancer on childbearing is largely confined to the early post‐diagnosis period.

**TABLE 2:** Cause‐specific hazard ratios (HR) and corresponding 95% confidence intervals (CI) of childbearing according to cancer diagnosis in reproductive age, overall, and in the subsets of the most common cancer types.

	Overall		Age at cancer diagnosis					
			15–29		30–39		≥40	
	Childbearing, *n* (%)^a^	HR (95% CI)	Childbearing, *n* (%)^a^	HR (95% CI)	Childbearing, *n* (%)^a^	HR (95% CI)	Childbearing, *n* (%)^a^	HR (95% CI)
**Overall**								
Matched cancer‐free	6824 (10.1)	Ref.	1704 (23.0)	Ref.	4532 (20.8)	Ref.	588 (1.6)	Ref.
Cancer survivors	899 (6.5)	0.66 (0.62–0.71)	308 (20.6)	0.93 (0.83–1.05)	532 (11.9)	0.86 (0.77–0.97)	59 (0.8)	0.58 (0.53–0.64)
**Breast cancer**								
Matched cancer‐free	1922 (6.9)	Ref.	196 (36.2)	Ref.	1451 (18.3)	Ref.	275 (1.4)	Ref.
Cancer survivors	166 (2.9)	0.41 (0.35–0.48)	27 (24.8)	0.67 (0.45–1.01)	117 (7.2)	0.37 (0.31–0.45)	22 (0.5)	0.40 (0.26–0.62)
**Thyroid cancer**								
Matched cancer‐free	1298 (14.0)	Ref.	396 (23.4)	Ref.	825 (22.5)	Ref.	77 (2.0)	Ref.
Cancer survivors	287 (15.1)	1.08 (0.95–1.23)	104 (30.5)	1.28 (1.03–1.59)	164 (21.8)	0.97 (0.82–1.15)	19 (2.3)	1.26 (0.76–2.08)
**Lymphoma**								
Matched cancer‐free	779 (17.1)	Ref.	348 (19.6)	Ref.	401 (25.2)	Ref.	30 (2.5)	Ref.
Cancer survivors	133 (14.3)	0.84 (0.70–1.01)	69 (19.3)	1.02 (0.79–1.32)	61 (18.7)	0.72 (0.55–0.94)	3 (1.2)	0.51 (0.16–1.66)

^a^ The “Childbearing, *n* (%)” columns report the absolute number and proportion of women who gave birth after their cancer diagnosis date (for cancer survivors, *N* = 13 877) the diagnosis date of the matched case (for matched cancer‐free women *N* = 67 297).

Of the women diagnosed with cancer, 7232 (52.1%) received antineoplastic therapy. The percentage of women treated with antineoplastic therapy was 64.2%, 3.6%, and 86.4% for breast, thyroid, and lymphoma cancers, respectively.

Further, women undergoing antineoplastic therapy were significantly less likely to give birth after their cancer diagnosis (4.0%) than those not receiving such treatment (9.0%, *p* < 0.01). The HR of 0.46 (95% CI: 0.39–0.52) underscores the substantial impact of antineoplastic therapy on fertility (Table 3). Stratified analysis by age at diagnosis yielded consistent results, with a higher impact observed at increasing age. The HRs for childbearing were 0.62 (95% CI: 0.49–0.79) among women diagnosed before the age of 30, 0.41 (95% CI: 0.34–0.49) among those diagnosed between 30 and 39 years, and 0.25 (95% CI: 0.13–0.47) among women diagnosed at 40 years or over. Table 3 also provides HRs of having a birth in women with cancer according to antineoplastic treatment in the subsets of the most common cancer types. Only for breast cancer, the probability of having a birth remained significantly lower in women who received antineoplastic treatment (HR = 0.42, 95% CI: 0.31–0.57); for thyroid cancer (HR = 1.10, 95% CI: 0.57–2.15) and lymphoma (HR = 0.83, 95% CI: 0.50–1.39), there was no association.

**TABLE 3:** Cause‐specific hazard ratios (HR) and corresponding 95% confidence intervals (CI) of childbearing in women with cancer diagnosis in reproductive age according to antineoplastic treatment, overall and in the subsets of the most common cancer types.

Antineoplastic treatment	Overall		Age at cancer diagnosis					
			15–29		30–39		≥40	
	Childbearing, *n* (%)^a^	HR (95%CI)	Childbearing, *n* (%)^a^	HR (95%CI)	Childbearing, *n* (%)^a^	HR (95%CI)	Childbearing, *n* (%)^a^	HR (95%CI)
**Overall**								
No	622 (9.4)	Ref.	203 (26.5)	Ref.	372 (17.7)	Ref.	47 (1.3)	Ref.
Yes	277 (3.8)	0.46 (0.39–0.52)	105 (14.4)	0.62 (0.49–0.79)	160 (6.7)	0.41 (0.34–0.49)	12 (0.3)	0.25 (0.13–0.47)
**Breast cancer**								
No	76 (3.7)	Ref.	8 (34.8)	Ref.	53 (12.5)	Ref.	15 (0.9)	Ref.
Yes	90 (2.4)	0.42 (0.31–0.57)	19 (22.1)	0.48 (0.21–1.10)	64 (5.3)	0.40 (0.28–0.58)	7 (0.3)	0.30 (0.12–0.75)
**Thyroid cancer**								
No	278 (15.1)	Ref.	101 (30.1)	Ref.	158 (21.8)	Ref.	19 (2.4)	Ref.
Yes	9 (13.2)	1.10 (0.57–2.15)	3 (60.0)	1.67 (0.53–5.27)	6 (22.2)	1.09 (0.48–2.46)	0 (0.0)	NA
**Lymphoma**								
No	17 (13.5)	Ref.	7 (28.0)	Ref.	9 (16.4)	Ref.	1 (2.2)	Ref.
Yes	116 (14.5)	0.83 (0.50–1.39)	62 (18.6)	0.72 (0.33–1.59)	52 (19.2)	0.93 (0.46–1.89)	2 (1.0)	0.46 (0.04–5.05)

^a^ The “Childbearing, *n* (%)” columns report the absolute number and proportion of women who gave birth after their cancer diagnosis, according to whether they received antineoplastic treatment or not.

Among the women who had a birth after their cancer diagnosis, 98 (10.9%) conceived through medically assisted reproduction. This proportion varied by cancer site; for instance, assisted reproduction was used in 21.2% of births after breast cancer, 12.7% of births after lymphoma, and 9.6% of births after thyroid cancer. The treatment with antineoplastic therapy was associated with the use of medically assisted reproduction to achieve a birth after cancer diagnosis (RR = 1.71, 95% CI: 1.14–2.56).

## 4 DISCUSSION

During the study period in Lombardy, almost 14,000 women of reproductive age were diagnosed with cancer. The most common type of cancer varied by age, with lymphoma more common in younger women and breast cancer at a later age. More than half of the women received antineoplastic therapy, with significant differences depending on the cancer type. The probability of having a birth decreased with increasing age at cancer diagnosis and, especially for breast cancer, with antineoplastic treatment. Overall, among younger cancer survivors, the likelihood of childbirth did not differ significantly from that of cancer‐free women. However, the probability of childbearing was reduced within the first 5 years of diagnosis, regardless of age at diagnosis. A more pronounced reduction was observed in women diagnosed after the age of 30. Beyond this period, no significant differences were observed compared to cancer‐free women.

As the age at first birth continues to rise in the general population,^10^ and a considerable fraction of women diagnosed with cancer in their childbearing years have not started or completed their families, there are concerns about subsequent fertility. Loss of menstrual function and lack of fertility options are emotionally distressing.^11^, ^12^ Infertility or subfertility is more common in cancer survivors than in healthy women.^13^ It is still unclear whether specific cancer types and stages^14^, ^15^ impact ovarian function, and hence ovarian response and the possibility of harvesting oocytes before treatment.^16^

According to our findings, age played an important role in determining the probability of cancer survivors having children. Older cancer survivors showed a significant reduction in the probability of childbearing, whereas younger women were only marginally affected. This association may be partly explained by differences in parity, as older women are more likely to already have children. One might speculate that women who have already had one child may feel a sense of fulfillment in their reproductive goals, reducing the motivation to have more children, regardless of their cancer history. In addition, the experience of cancer itself may lead to a reassessment of personal priorities, which could further influence the decision not to have more children, especially if they have already had children, even among those who remain biologically capable. However, as acknowledged in the limitations of our study, this aspect was not explicitly investigated and further research is needed to assess its potential impact.

Importantly, the temporal pattern of reduced childbearing concentrated in the years immediately following diagnosis suggests that cancer and its treatment have the greatest impact on fertility shortly after diagnosis. From a clinical perspective, many oncological treatments are acutely gonadotoxic and may impair ovarian function. However, this impairment may be partial and is sometimes reversible.^17^ Additionally, prolonged adjuvant endocrine therapies, such as tamoxifen for breast cancer, can delay attempts to conceive by several years.^18^ Psychosocially, many women deliberately postpone childbearing in order to focus on treatment and recovery. This decision is often influenced by fears of recurrence, concerns about long‐term prognosis, and worries about the potential impact of prior cancer treatment on the health of any future children. Furthermore, cancer can result in economic or relational instability, which can further discourage short‐term reproductive planning.

The distribution of cancer sites also differed by age. In particular, thyroid cancer is more commonly diagnosed in younger women and is typically associated with less frequent antineoplastic treatment and a more favorable prognosis.^19^ This appears consistent with the higher childbearing rates observed in women diagnosed with thyroid cancer in our study, especially in younger women.

A cancer diagnosis in a young woman raises concerns about ovarian function and potential infertility, and influences attitudes and decisions about pregnancy, breastfeeding, and contraception. Thus, the need for tailored fertility‐related information for adolescents and young adults diagnosed with cancer has been advocated.^8^

A common cause of female infertility is the reduction of ovarian reserve. The ovary is vulnerable to the direct and indirect effects of chemotherapy, and the risk of treatment‐induced gonadotoxicity is one of the most concerning short‐ and long‐term side effects of cancer therapy.^20^ The germ cell pool can be damaged either through a direct effect on double‐strand DNA or by accelerating follicular activation,^21^ but also indirectly by affecting the stroma, leading to a decrease in blood vessels and a reduction in blood supply.^22^ The risk of ovarian failure depends not only on age and other patient characteristics, but also on the type of drugs used and the dose administered.^23^, ^24^ For example, alkylating agents such as cyclophosphamide have been associated with high gonadotoxic impact,^25^ whereas there is limited data on the potential gonadotoxic effects of new drugs such as monoclonal antibodies or kinase inhibitors.^26^ The use of gonadotropin‐releasing hormone agonist (GnRHa) as part of treatment to prevent chemotherapy‐induced premature ovarian failure has been studied in particular in breast cancer patients, and has been shown to reduce the risk regardless of hormone receptor status.^26^ In line with this, we found that the likelihood of live birth was lower in women diagnosed with cancer at the age of 35 or older, and halved in those who had received antineoplastic treatment.

Gonadal function is also adversely affected by radiotherapy. The dose, field of radiation, and age of the patient determine the degree and persistence of damage. In particular, radiotherapy for pelvic and abdominal diseases, such as cervical and rectal cancer or lymphomas with pelvic lymph node involvement, and spinal radiotherapy for central nervous system tumors, exposes the ovaries to substantial doses of radiation.^27^

Reduced fertility affects up to 80% of survivors, and about 70%–75% of young cancer survivors are interested in becoming parents.^28^ We found that 11% of women who gave birth after cancer underwent assisted reproduction, compared to about 3% in all births in Lombardy between 2007 and 2020.^29^ In addition, antineoplastic therapy was associated with assisted reproduction, with a twofold frequency.

Appropriate oncofertility counseling for all premenopausal women, regardless of the type of disease and stage, is recommended.^23^, ^24^ During counseling, patients should be informed about the risk of premature ovarian failure as a result of the proposed cancer treatment and about the strategies available to counteract this side effect. Counselors should explain the differences between fertility preservation and ovarian function preservation, as well as the criteria for inclusion in different strategies. Fertility preservation should be discussed with any patient whose fertility may be affected by medical treatment or disease.^30^, ^31^ According to the European Society for Medical Oncology,^23^ recommendations for female fertility preservation offer alternatives based on several parameters (e.g., the age of the patient, cancer site, treatment, presence or absence of a partner), including embryo, unfertilised oocyte, and ovarian tissue cryopreservation, ovarian transplantation, and ovarian suppression.

For ovarian function preservation, the only recommended medical treatment is the administration of GnRHa during chemotherapy. As mentioned above, this aims to reduce the risk of chemotherapy‐induced ovarian damage and prevent premature ovarian insufficiency in premenopausal women, including those over the age of 40 who are typically not candidates for fertility preservation. In fact, GnRHa is a suitable option not only for women concerned about preserving their fertility, but also for those who want to avoid the negative effects of premature ovarian insufficiency.^32^ The long‐term impact of estrogen deficiency due to early loss of ovarian function on quality of life and overall health should not be underestimated.^33^

A recent study of women's experiences of the perinatal period after cancer found that women experience a duality of feelings during the perinatal period that is driven by their cancer history, captured by the expression: “*I'm so happy, but also terrified.”* ^34^ Many women who have had cancer, especially hormone‐sensitive cancers such as breast cancer,^35^ may be concerned that the hormonal changes during pregnancy could trigger a recurrence. Evidence generally suggests that pregnancy,^36^ and also breastfeeding,^37^ does not increase the risk of breast cancer recurrence or second breast cancer. In women with BRCA alterations, the fear of developing other cancer may also reduce their desire for children.^38^

The population‐based design is a major strength of this study. The automated system of NHS databases represents large and validated data sources that cover patient information over a 10‐year period. Among the possible limitations, our definition of a cancer diagnosis was based on a hospital admission for a malignant disease. However, if the diagnosis was made in an outpatient setting, hospital data may be less efficient in determining the exact time of diagnosis. To avoid misclassifying treatment status due to potential delays between outpatient diagnosis and hospital admission, we considered all women who received antineoplastic therapy within a year of the hospitalization date used for cancer diagnosis to have been treated. In the main analysis, considering all neoplasms together may be a limitation, as different cancers have different courses and effects on fertility outcomes. Therefore, we also performed stratified analyses for the three most common cancers, even though the number of cases within each cancer site group was limited, which potentially reduced the statistical power of these analyses. Additionally, we lacked information about the stage of the disease. Another lacking piece of information was parity, as we were unable to reconstruct the full obstetric history for all women included in the analysis. This prevents us from fully accounting for the potential impact of parity on desire and thus the likelihood of pregnancy after cancer diagnosis. Finally, when we evaluated the mode of conception, we did not know whether those who had undergone assisted reproduction had used gamete preservation.

## 5 CONCLUSION

Our findings suggest that age and exposure to antineoplastic therapy are critical determinants of childbearing in women with a cancer diagnosis. While younger survivors appear to retain similar childbearing potential compared to cancer‐free counterparts, older women show a notable decline.

## AUTHOR CONTRIBUTIONS

Conceptualization: CLV, FP, EN; methodology: AC, GE, MF; formal analysis and data curation: AC, GE, MF; investigation: AC, GE, MF; writing—original draft preparation: GE; writing—review and editing: all authors; supervision: GC, CLV, FP, EN; all authors have read and approved the final version of the manuscript.

## FUNDING INFORMATION

This work is supported by the European Union—Next Generation EU, Mission 4, Component 1, CUP: H53D23006310001 (PRIN 2022. Real‐world evaluation of cancer outcomes by integrating administrative and hospital‐based health‐related data: the “We‐Care” project).

## CONFLICT OF INTEREST STATEMENT

Prof Giovanni Corrao received research support from the European Community (EC), the Italian Agency of Drugs (AIFA), and the Italian Ministry for University and Research (MIUR). He took part in a variety of projects that were funded by pharmaceutical companies (i.e., Novartis, GSK, Roche, AMGEN, and BMS). He also received honoraria as a member of the advisory board to Roche. The other authors declare no conflict of interests.

## ETHICS STATEMENT

Data used in this study were anonymized before their use. According to Italian law, studies based entirely on registry data are exempt from IRB authorization and informed consent.

## Supporting information


**Table S1.** Cause‐specific hazard ratios (HR) and corresponding 95% confidence intervals (CI) of childbearing according to cancer diagnosis in reproductive age, overall, and in the subsets of the most common cancer types, stratified by time since diagnosis (i.e., ≤5 and >5 years).

## Data Availability

The data that support the findings of this study are available from Lombardy region. Restrictions apply to the availability of these data, which were used under license for this study. Data are available from the author(s) with the permission of Lombardy region.
