# Supplementary material for: Childbearing in women diagnosed with cancer during reproductive age
Source: Acta Obstet Gynecol Scand. 2025 Oct 27;104(12):2309–19. doi: 10.1111/aogs.70071 (PMC12668817; doi:10.1111/aogs.70071)
Supplement: Supplementary file 1 — Table S1. Cause‐specific hazard ratios (HR) and corresponding 95% confidence intervals (CI) of childbearing according to cancer diagnosis in reproductive age, overall, and in the subsets of the most common cancer types, stratified by time since diagnosis (i.e., ≤5 and >5 years). [file AOGS-104-2309-s001.docx]

**Table S1.** Cause-specific hazard ratios (HR) and corresponding 95% confidence intervals (CI) of childbearing according to cancer diagnosis in reproductive age, overall, and in the subsets of the most common cancer types, stratified by time since diagnosis (i.e., ≤5 years and >5 years).

|  |  | **Overall** | | **Age at cancer diagnosis** | |  | |  | |
| --- | --- | --- | --- | --- | --- | --- | --- | --- | --- |
|  |  |  |  | **15-29** | | **30-39** | | **≥40** | |
|  |  | **Childbearing, n (%)** | **HR (95%CI)** | **Childbearing, n (%)** | **HR (95%CI)** | **Childbearing, n (%)** | **HR (95%CI)** | **Childbearing, n (%)** | **HR (95%CI)** |
|  |  |  |  |  |  |  |  |  |  |
| **Within the 5 years after the diagnosis** | *Overall* |  |  |  |  |  |  |  |  |
|  | Matched cancer-free | 5441 (8.1) | Ref. | 1001 (13.5) | Ref. | 3884 (17.8) | Ref. | 556 (1.5) | Ref. |
|  | Cancer survivors | 612 (4.4) | 0.59 (0.54-0.64) | 153 (10.2) | 0.82 (0.69-0.97) | 407 (9.1) | 0.55 (0.49-0.61) | 52 (0.7) | 0.49 (0.37-0.67) |
|  | *Breast cancer* |  |  |  |  |  |  |  |  |
|  | Matched cancer-free | 1630 (5.8) | Ref. | 116 (21.4) | Ref. | 1251 (15.8) | Ref. | 263 (1.4) | Ref. |
|  | Cancer survivors | 96 (1.7) | 0.29 (0.23-0.36) | 9 (8.3) | 0.41 (0.21-0.81) | 70 (4.3) | 0.27 (0.21-0.35) | 17 (0.4) | 0.34 (0.20-0.56) |
|  | *Thyroid cancer* |  |  |  |  |  |  |  |  |
|  | Matched cancer-free | 1014 (10.9) | Ref. | 233 (13.8) | Ref. | 711 (19.4) | Ref. | 70 (1.8) | Ref. |
|  | Cancer survivors | 194 (10.2) | 0.97 (0.83-1.14) | 39 (11.4) | 0.86 (0.61-1.21) | 137 (18.2) | 0.98 (0.81-1.18) | 18 (2.2) | 1.35 (0.78-2.35) |
|  | *Lymphoma* |  |  |  |  |  |  |  |  |
|  | Matched cancer-free | 564 (12.4) | Ref. | 191 (10.7) | Ref. | 347 (21.8) | Ref. | 26 (2.2) | Ref. |
|  | Cancer survivors | 92 (9.9) | 0.85 (0.68-1.06) | 42 (11.7) | 1.14 (0.81-1.60) | 47 (14.4) | 0.70 (0.51-0.95) | 3 (1.2) | 0.66 (0.20-2.20) |
|  |  |  |  |  |  |  |  |  |  |
| **Beyond the 5 years after the diagnosis** | *Overall* |  |  |  |  |  |  |  |  |
|  | Matched cancer-free | 1383 (2.3) | Ref. | 703 (11.3) | Ref. | 648 (3.7) | Ref. | 32 (0.1) | Ref. |
|  | Cancer survivors | 287 (2.5) | 1.05 (0.92-1.19) | 155 (13.2) | 1.15 (0.97-1.37) | 125 (3.7) | 0.97 (0.80-1.18) | 7 (0.1) | 1.21 (0.53-2.74) |
|  | *Breast cancer* |  |  |  |  |  |  |  |  |
|  | Matched cancer-free | 292 (1.1) | Ref. | 80 (19.6) | Ref. | 200 (3.1) | Ref. | 12 (0.1) | Ref. |
|  | Cancer survivors | 70 (1.3) | 1.10 (0.85-1.43) | 18 (20.7) | 1.08 (0.65-1.80) | 47 (3.4) | 1.09 (0.79-1.50) | 5 (0.1) | 2.11 (0.74-6.00) |
|  | *Thyroid cancer* |  |  |  |  |  |  |  |  |
|  | Matched cancer-free | 284 (3.5) | Ref. | 163 (11.6) | Ref. | 114 (3.9) | Ref. | 7 (0.2) | Ref. |
|  | Cancer survivors | 93 (5.6) | 1.59 (1.26-2.00) | 65 (22.1) | 1.98 (1.48-2.64) | 27 (4.5) | 1.14 (0.75-1.74) | 1 (0.1) | 0.70 (0.09-5.66) |
|  | *Lymphoma* |  |  |  |  |  |  |  |  |
|  | Matched cancer-free | 215 (5.5) | Ref. | 157 (10.2) | Ref. | 54 (4.5) | Ref. | 4 (0.4) | Ref. |
|  | Cancer survivors | 41 (5.3) | 0.94 (0.67-1.32) | 27 (9.1) | 0.91 (0.61-1.37) | 14 (5.5) | 1.21 (0.67-2.17) | 0 (0.0) | NA |
|  |  |  |  |  |  |  |  |  |  |

The "Childbearing, n (%)" columns report the absolute number and proportion of women who gave birth after their cancer diagnosis date (for cancer survivors) the diagnosis date of the matched case (for matched cancer-free women).
